# Integrin β3-Mediated Cell Senescence Associates with Gut Inflammation and Intestinal Degeneration in Models of Alzheimer’s Disease

**DOI:** 10.3390/ijms24065697

**Published:** 2023-03-16

**Authors:** Xin Tun, Evan J. Wang, Zhenxiang Gao, Kathleen Lundberg, Rong Xu, Di Hu

**Affiliations:** 1 Department of Physiology and Biophysics, Case Western Reserve University School of Medicine, Cleveland, OH 44106, USA; 2 Center for Artificial Intelligence in Drug Discovery, Case Western Reserve University School of Medicine, Cleveland, OH 44106, USA; 3 Beachwood High School, Beachwood, OH 44122, USA; 4 Proteomics Center, Case Western Reserve University School of Medicine, Cleveland, OH 44106, USA

**Keywords:** Alzheimer’s disease, colon, epithelial senescence, inflammation, integrin β3

## Abstract

Alzheimer’s disease (AD) is a neurodegenerative disorder characterized by memory loss and personality changes that ultimately lead to dementia. Currently, 50 million people worldwide suffer from dementia related to AD, and the pathogenesis underlying AD pathology and cognitive decline is unknown. While AD is primarily a neurological disease of the brain, individuals with AD often experience intestinal disorders, and gut abnormalities have been implicated as a major risk factor in the development of AD and relevant dementia. However, the mechanisms that mediate gut injury and contribute to the vicious cycle between gut abnormalities and brain injury in AD remain unknown. In the present study, a bioinformatics analysis was performed on the proteomics data of variously aged AD mouse colon tissues. We found that levels of integrin β3 and β-galactosidase (β-gal), two markers of cellular senescence, increased with age in the colonic tissue of mice with AD. The advanced artificial intelligence (AI)-based prediction of AD risk also demonstrated the association between integrin β3 and β-gal and AD phenotypes. Moreover, we showed that elevated integrin β3 levels were accompanied by senescence phenotypes and immune cell accumulation in AD mouse colonic tissue. Further, integrin β3 genetic downregulation abolished upregulated senescence markers and inflammatory responses in colonic epithelial cells in conditions associated with AD. We provide a new understanding of the molecular actions underpinning inflammatory responses during AD and suggest integrin β3 may function as novel target mediating gut abnormalities in this disease.

## 1. Introduction

As a prevalent neurodegenerative disease, Alzheimer’s disease (AD) is categorized by learning disabilities, personality changes, memory loss, and finally culminates in dementia [1]. Globally, more than 46.8 million individuals have AD-related dementia, and by 2050, this number is anticipated to increase to 131.5 million [2,3]. In recent decades, despite advances in our understanding of AD pathogenesis, cognitive deficit and cell damage mechanisms associated with AD remain unclear, while effective treatments preventing or limiting AD progression are unknown.

While AD is primarily a neurological disease of the brain, individuals with AD often experience debilitating gastrointestinal tract (GIT) disorders, including fecal incontinence, constipation, intestinal volvulus, and colon dilatation [4,5]. Both patients with AD and AD experimental models experience altered gut microbiome composition, which is related to significant pro-inflammatory cytokine gene expression profiles [6,7,8,9]. Notably, individuals with abnormal GIT disorders show rapid cognitive decline [10], while antibiotic-mediated perturbations in the gut microbiome can modulate dementia onset and amyloid accumulation in AD animals [11,12]. Recently, a longitudinal study reported that individuals with inflammatory bowel disease had more than a six-fold increased risk of AD and a possible five-fold increased incidence of all dementia types [13]. Furthermore, recent genetic data also suggested that GIT disorders shared common genetic causes with AD and were considerable AD risk factors [14]. Additionally, some gut microbiota bacterial products, such as short-chain fatty acid metabolites, induced neuroinflammation, increased amyloid plaque deposition, and promoted cognitive alterations in transgenic AD mice [9,15]. Similarly, gut microbiota removal was also reported to reduce neuropathology in transgenic AD mice [9,16]. Despite increasing investigations showing that gut abnormalities are implicated in AD etiology [17,18], the signals that induce gut injury and promote the vicious cycle between gut abnormalities and brain injury in AD remain unknown.

The permanent loss of somatic cell proliferative potential, thereby preventing damaged cell propagation, is called cell senescence. The process is characterized by chromatin remodeling, DNA damage, cell cycle inhibitor pathway (p21/p53 and/or p16/pRb) activation, senescence-associated secretory phenotype (SASP) development, and senescence-associated β-galactosidase (SA-β-Gal) activity [19,20]. Under normal conditions, different inflammatory proteins and metalloproteases which comprise SASP’s are secreted from senescent cells, after which SASP’s activate immune responses to eliminate senescent cells and promote tissue homeostasis [19]. However, during aging and age-related diseases, e.g., AD, senescent cells accumulate during disease progression and induce severe SASPs, causing inflammatory responses, cytokine accumulation, and increased reactive oxygen species which degenerate tissue [21]. Thus, senescent cell depletion in aging tissue may limit age-related pathological progress and severity, and even ameliorate lifespan [21,22]. In previous studies, senescent cell removal using pharmacological or genetic approaches has reversed senescent phenotypes, suppressed inflammation, and attenuated cognitive deficits and neuronal loss in different animal AD models [23,24,25].

During aging, the GIT epithelium becomes more permeable due to proinflammatory cytokine release, which increases gut permeability and alters gut microbiota composition [26,27]. Recently, age-dependent increases in senescent p21^+^ cells were observed in different human organs, including colon tissue, and indicated associations between senescent cell accumulation and intestinal aging [28]. In particular, the cellular senescence signatures were identified in intestinal tissue via elevated p21 levels and SA-β-gal activity in accelerated aging and wild-type (WT) mouse models [29]. Similarly, in aged mice, these senescence markers were upregulated in intestinal epithelial organoids when compared with younger mice [30]. Therefore, intestinal epithelial cells exhibit age-dependent cell senescence, which may be implicated in gut homeostasis disruption and intestinal inflammation. However, in pathophysiological terms, it is unclear if cell senescence occurs in the intestines of mice with AD and associates with AD development.

In this study, we investigated age-dependent degeneration in the intestinal tissue of transgenic AD mice. Unbiased proteomic and biochemical analyses showed that integrin β3 and β-galactosidase (β-gal), two markers implicated in cellular senescence, were increased with age in AD mouse colonic tissue. Elevated integrin β3 levels were accompanied by increased p21 and SA-β-gal activity, induced SASPs, and accumulated inflammatory cell infiltration in AD mice. Integrin β3 was highly enriched in intestinal epithelial cells and its elevation induced cell senescence signatures in colonic epithelial cells. Notably, the genetic downregulation of integrin β3 abolished upregulated cell senescence markers and inflammatory responses in colonic epithelial cells under AD conditions. Our results suggest that integrin β3-mediated intestinal cell senescence may represent a molecular signal that triggers intestinal inflammation and tissue degeneration in AD, thereby contributing to AD progression.

## 2. Results

### 2.1. Age-Dependent Intestinal Tissue Degeneration in Mouse AD Models

While previous studies have reported intestinal microbiota alteration associations with AD development [5,7,17,26], it currently remains unclear how intestinal tissue integrity changes occur in line with disease progression. Colon dilatation is correlated with human AD [4,5], and 5XFAD mouse colons appear to be most affected by AD stimuli [31]. Therefore, we first characterized colon enteric network density in 5XFAD mice at 3- (early pathological stage), 6- (advanced pathological stage), and 9-months-old (late pathological stage). Transgenic 5XFAD mice have five AD mutations which increase Aβx-42 levels and accelerate plaque formation. Using site-directed mutagenesis, the model was formed by introducing amyloid precursor proteins (APPs) of the Swedish, Florida, and London mutations, and also PS1 L286V and M146L mutations into APP (695) and PS1 cDNAs [32]. We used this model as APP is expressed in human AD and mouse AD model guts [11,33,34,35,36] and several AD traits are present in the 5XFAD model, including gut alterations [11,31,32].

**Figure 1:**
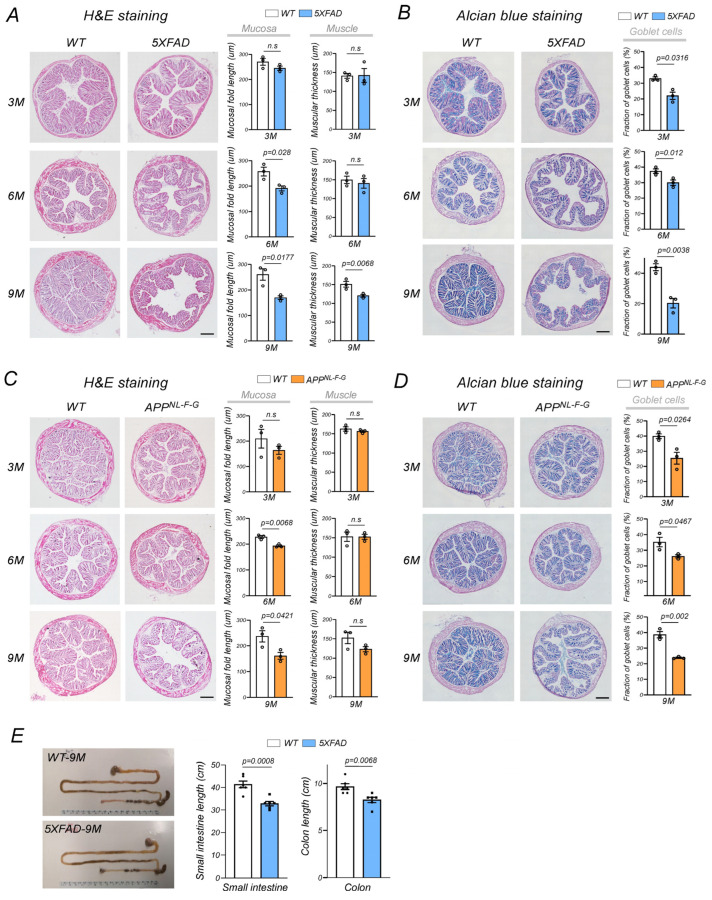
Colonic abnormalities in AD mouse models. (**A**) Hematoxylin and eosin (H&E) staining of colonic wild-type (WT) and 5XFAD mouse sections at 3- (3M), 6- (6M), and 9-months-old (9M). Cross-sectional mucosal fold lengths and muscular thickness were measured and quantified as shown (*n* = 3 mice/group. Scale bar = 300 μm). (**B**) Alcian blue staining of goblet cells in WT and 5XFAD mouse colons at different ages (*n* = 3 mice/group. Scale bar = 300 μm). (**C**) H&E staining of WT and APP^NL-F-G^ colonic sections at different ages. Cross-sectional mucosal fold length and muscular thickness were measured and quantified as shown in histograms (*n* = 3 mice/group. Scale bar = 300 μm). (**D**) Alcian blue staining of goblet cells in WT and APP^NL-F-G^ mouse colons at different ages (*n* = 3 mice/group. Scale bar = 300 μm). (**E**) Intact guts were isolated from WT and 5XFAD mice when 9-months-old, and small intestine and colon lengths measured. Results are shown (*n* = 6/group). Data were expressed as the mean ± standard error of the mean and compared using unpaired Student’s *t*-tests. n.s: no significance.

From H&E staining, age-dependent decreases in the cross-sectional length of colon mucosal folds were identified in 5XFAD mice relative to age-matched WT controls (Figure 1A). While colon muscular thickness was similar between all mice at 3- and 6-months-old, significant decreases in thickness were observed in 9-month-old 5XFAD animals (Figure 1A). Using Alcian blue staining, we consistently observed age-dependent intestinal content loss in 5XFAD mice when compared with age-matched WT controls. Alcian blue-stained goblet cell fractions were progressively shortened in 5XFAD mouse colons and showed approximately 50% loss at 9-months-old (Figure 1B), which is consistent with goblet cell degeneration in human AD gut tissues [37]. To exclude the possibility that enteric network density loss was caused by APP overexpression in 5XFAD mice, we examined colon structures in APP^NL-F-G^ knock-in (KI) mice which expressed human AD mutations at a humanized mouse APP locus; mutations were driven by the APP endogenous promoter, therefore animals developed neurocognitive defects and amyloid plaques [38]. This KI construct had a humanized Aβ region and three pathogenic Swedish “NL”, Iberian “F”, and Arctic “G” mutations [38]. Mice expressed endogenous APP levels and avoided possible artifacts mediated by APP overexpression while simultaneously generating increased pathogenic Aβ levels [38]. In our study, these KI mice exhibited age-dependent colon mucosal fold and goblet cell degeneration, with severe loss at 9-months-old (Figure 1C,D). Additionally, the overall small intestine and colon length in 5XFAD mice was significantly shorter when compared with age-matched WT controls (Figure 1E). Collectively, we observed age-dependent intestinal tissue degeneration in APP-associated mouse AD models.

### 2.2. Global Colon Protein Expression Features in 5XFAD Mice across Ages

**Figure 2:**
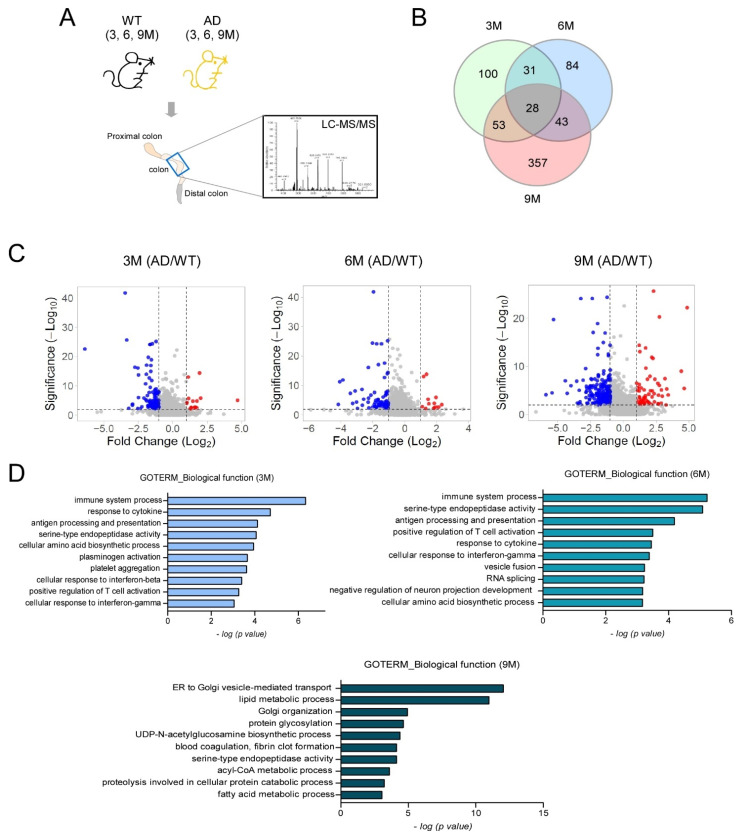
Colonic protein profiling alterations in AD mice over time. Colons were harvested from 5XFAD and WT mice at 3, 6, and 9 months and label free LC-MS/MS analyses were performed. (**A**) Proteomics analysis scheme. (**B**) The Venn diagram shows the proteins that were altered in 5XFAD colons over time (5XFAD/WT: 0.5 ≥ fold change ≥ 2). (**C**) Volcano plots represent significantly altered proteins (blue = down-regulated and red = up-regulated) in 5XFAD mice at 3, 6, and 9 months (5XFAD/WT: 0.5 ≥ fold change ≥ 2, unpaired *t*-test). (**D**) Gene Ontology biological process pathway analyses on altered proteins in 5XFAD mouse colons at 3, 6, and 9 months (Fischer’s exact test, *p* < 0.01). The top 10 pathways at each time point are shown.

To investigate the molecular basis underlying intestinal tissue damage in AD mice, we profiled the proteome in 5XFAD mouse colonic tissue at different times to reflect disease progression. Proximal and distal colonic tissue were harvested from 5XFAD mice at 3-, 6-, and 9-months-old, and label-free LC-MS/MS analyses were conducted (Figure 2A). Colonic tissues from age-matched WT controls served as comparisons. From these analyses, proteins with at least a two-fold expression change in 5XFAD versus WT controls were selected (i.e., >2-fold down- or up-regulation relative to WT animals). Using these criteria, 214, 88, and 483 proteins were identified in 3-, 6-, and 9-month-old 5XFAD mouse colonic tissue datasets, respectively (Figure 2B). The volcano plots for each dataset highlighted a dramatic accumulation of significantly differentially expressed proteins with progression of time (5XFAD/WT 0.5 ≥ FC ≥ 2, depicted as blue and red dots) (Figure 2C). At 3, 6, and 9 months, 64, 42, and 161 proteins were upregulated, while 150, 46, and 322 proteins were downregulated, respectively (Figure 2C). To examine biological pathways which were transiently associated with AD, separate enrichment pathway analyses were exclusively performed for proteins dysregulated at all times points. Similar pathways were also identified as significantly dysregulated at 3 and 6 months in 5XFAD mice; “immune system processes” (*p* = 4.5*10^−7^ at 3 months and *p* = 5.83*10^−6^ at 6 months) was ranked as the top enrichment pathway (Figure 2D). In the 9 month 5XFAD colon tissue dataset, vesicle-mediated transport (*p* = 8.53*10^−13^) and lipid metabolic processes (*p* = 9.82*10^−12^) were the top two enriched pathways (Figure 2D). Immune responses, vesicle transportation, and lipid metabolism mutually affect each other in AD [39,40,41]. This cross-sectional proteomic analysis suggested that 5XFAD mouse colonic tissue underwent time-sensitive and complex proteomic alterations during AD development, with striking shifts away from early immune response signatures at 3 and 6 months to dysregulated lipid metabolism at 9 months (Figure 2D).

**Figure 3:**
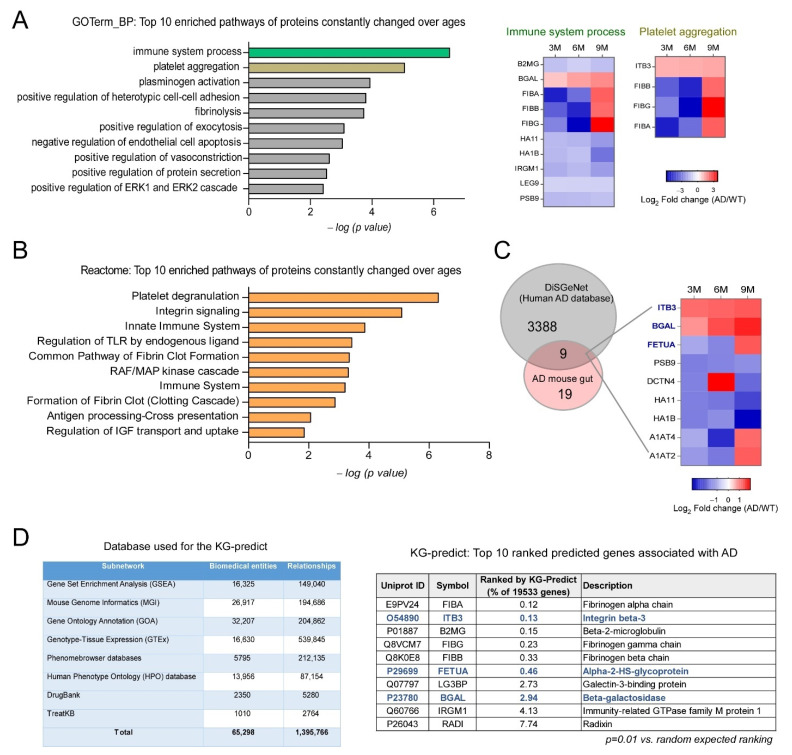
Tracking colonic proteins associated with AD concomitant with disease progression. From our cross-sectional study (3, 6, and 9 months), we analyzed 28 proteins which were consistently changed in 5XFAD mouse colons. (**A**) Top 10 pathways using gene ontology biological process analysis. Fischer’s exact test, *p* < 0.01. Right panel: altered proteins enriched in “immune system processes” and “platelet aggregation”. (**B**) Top 10 pathways from Reactome analysis. Fischer’s exact test, *p* < 0.01. (**C**) The Venn diagram shows overlaps between DiSGeNET’s AD and gene-disease association dataset (#C0002395) and proteins consistently altered in 5XFAD mouse colons over time. The heat map shows nine overlapped proteins that were altered in 5XFAD mouse colons over time. (**D**) Artificial intelligence-based KG prediction framework was used to predict the association of 28 consistently altered proteins in 5XFAD mouse colons with AD. Left: the table shows database construction which was used for KG predictions. Right: the table shows the top 10 ranked proteins associating with AD. KG predictions confirmed proteins which were known to associate with AD, and also predicted new proteins potentially associating with AD; ITGB3, PETUA, and BGAL (blue) are three proteins overlapping from C and D analyses.

Across time points, we identified 28 differentially expressed proteins (Figure 3). A graphical comparison of GO Biological Pathway terms on these proteins showed that “immune system processes”, “platelet aggregation”, and plasminogen activation” were the top three protein enrichment pathways (Figure 3A). Subsequent Reactome analyses identified “integrin signaling” as an additional top enrichment pathway (Figure 3B). We matched these 28 proteins to the DisGeNET AD biomarker database (CUI: C002395) and identified nine AD-associated protein biomarkers (ITGB3, βGal, FETUA, PSB9, DCTN4, HA11, HA1B, AiAT4, and A1AT2) (Figure 3C). To identify relationships between these 28 proteins and AD, we performed advanced artificial intelligence-based computational analyses (Figure 3). Using our KG-prediction approach [42,43], we ranked biomedical entities using our context-sensitive network-based algorithm. In total, 19,533 proteins were analyzed, and as shown (Figure 3D), the top ten predicted proteins from the 28 proteins that were significantly associated with AD (*p* ≤ 0.01 vs. random expected ranking) were ranked. Interestingly, three proteins (ITGB3, FETUA, and βGAL) that were significantly related to AD overlapped with proteins from the DisGeNET AD biomarker database (Figure 3D). Both ITGB3 and β-gal showed age-dependent increases in 5XFAD mouse colonic tissue (Figure 3). β-gal is a glycoside hydrolase enzyme used as a cell senescence marker [19,28]. Integrin β3 belongs to the integrin family and has key functions in migration, cell proliferation, immune responses, microenvironmental homeostasis via extracellular vesicles, and tissue repair [44,45]. The protein also regulates cell senescence by activating transforming growth factor-β signaling in an autocrine and paracrine fashion [46]. Thus, progressively increased integrin β3 and β-gal levels may implicate cell senescence in intestinal tissue damage in AD.

### 2.3. Inflammatory Cell Infiltration into the 5XFAD Mouse Colon

**Figure 4:**
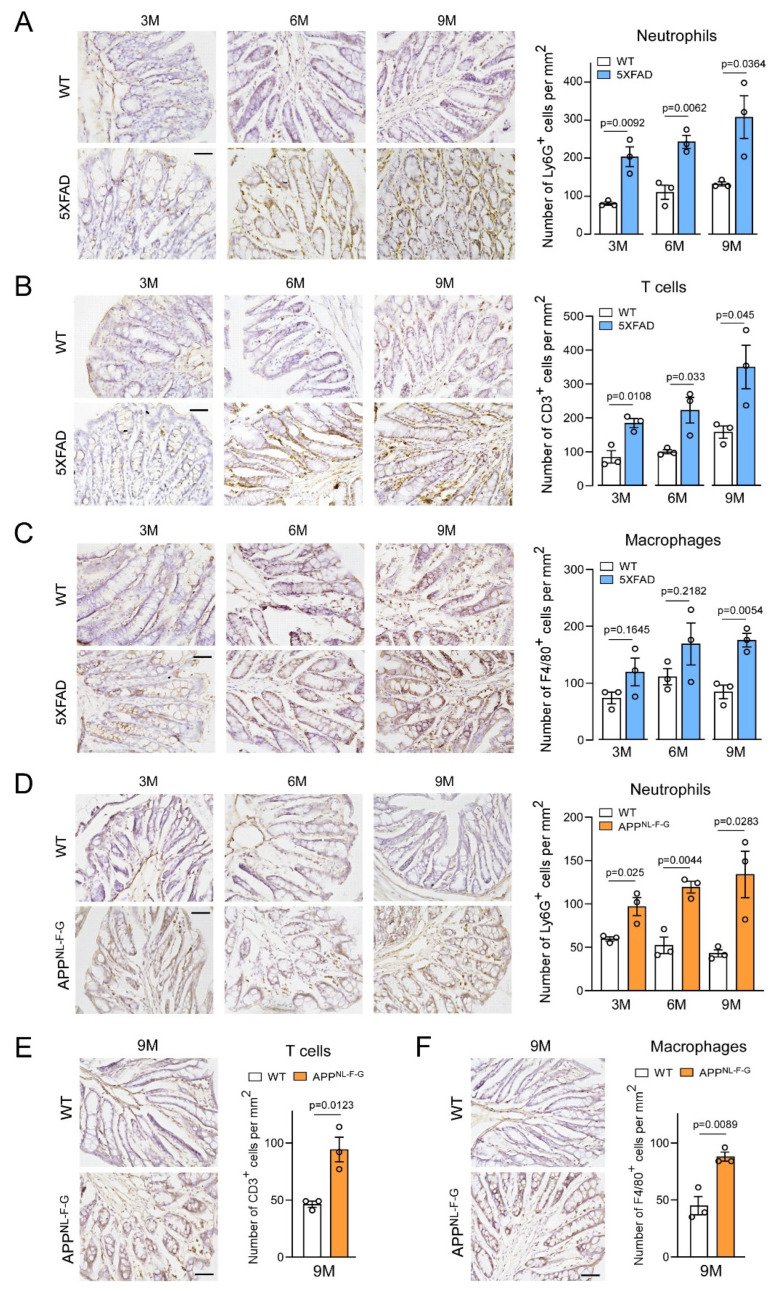
Immune cell infiltration into AD mouse model colons. (**A**) Neutrophil, (**B**) T cell, and (**C**) macrophage infiltration into WT and 5XFAD mouse colons at 3 (3M), 6 (6M), and 9 (9M) months were examined via the immunohistochemical staining of specific immune cell markers using anti-Ly6G, anti-CD3, and anti-F4/80 antibodies (*n* = 3 mice/group. Scale bar =20 μm). Similarly, immune cell infiltration into WT and APP^NL-F-G^ mouse colons at different ages were determined via immunohistochemical staining using anti-Ly6G, anti-CD3, and anti-F4/80 antibodies in (**D**), (**E**), and (**F**), respectively. (*n* = 3 mice/group. Scale bar = 20 μm). Quantified immune cell numbers/mm^2^ are shown. Data were expressed as the mean ± standard error of the mean and compared using unpaired Student’s *t*-tests.

Our cross-sectional proteomic analysis of 5XFAD mouse colons suggested that proteins enriched in immune response were mostly affected across different ages (Figure 3). We next examined if immune changes were associated with intestinal pathology in 5XFAD mice. 5XFAD and WT mouse colonic sections (all ages) were stained using anti-Ly6G (neutrophil marker), anti-CD3 (T cell marker), and anti-F4/80 (macrophage marker) antibodies. IHC analyses identified age-dependent increases in cells immune-positive for anti-Ly6G, CD3, or F4/80 antibodies in 5XFAD colon tissues starting at 3 months when compared with age-matched WT controls (Figure 4A–C). Age-dependent increases in Ly6G^+^, CD3^+^, and F4/80^+^ cells were consistently observed in APP^NL-F-G^ KI mouse colons (Figure 4D–F). Thus, inflammatory cell infiltration and accumulation, including neutrophils, T cells, and macrophages, had occurred in AD mouse colons, concomitant with intestinal tissue degeneration and AD progression. These findings in our mouse models were consistent with the observations that inflammatory cells were increased in human AD gut tissues [37], which may lead to gut barrier dysfunction [8]. Importantly, these findings supported our proteomic analyses showing that altered immune phenotypes characterized intestinal tissue in AD mice.

### 2.4. Increased Integrin β3 Levels Are Associated with Cell Senescence Signatures in 5XFAD Mouse Colons

**Figure 5:**
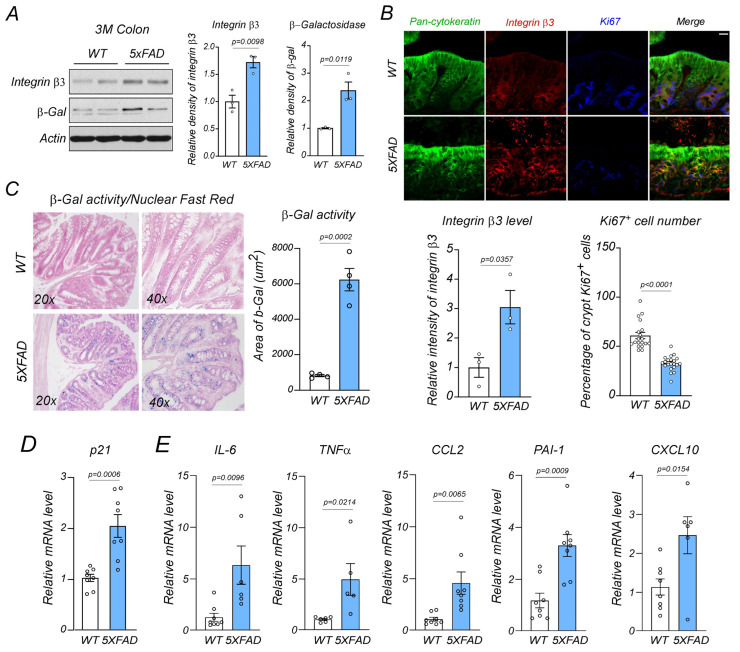
Integrin β3 upregulation and senescence in the AD mouse colon. (**A**) Integrin β3 and β-galactosidase (β-gal) protein levels were examined using western blotting in wild-type (WT) and 5XFAD mouse colons at 3 months (3M) (*n* = 3 mice/group). (**B**) Co-immunofluorescence staining of colon mucosal epithelium using anti-pan-cytokeratin, anti-integrin β3, and anti-Ki67 antibodies in WT and 5XFAD mice at 3 months. Relative integrin β3 immuno-intensity and Ki67^+^ cell percentages/colon crypt are shown (*n* = 3 mice/group. Scale bar = 25 μm). (**C**) β-galactosidase activity was determined via X-gal staining and followed by nuclear fast red staining in WT and 5XFAD mouse colonic sections at 3 months (*n* = 4 mice/group). (**D**) p21- and (**E**) senescence-associated secretory phenotype-related gene mRNA levels were determined via a RT-qPCR in WT and 5XFAD mouse colons at 3 months. (*n* = 8 mice/group). Data were expressed as the mean ± standard error of the mean and compared using unpaired Student’s *t*-tests.

Western blotting confirmed significant increases in integrin β3 and β-galactosidase protein levels in 5XFAD mouse colons (Figure 5A). Immunofluorescence staining also showed significant rises in integrin β3 expression levels in 5XFAD mice when compared with WT controls (Figure 5B). Additionally, increased integrin β3 immuno-intensity levels were observed in pan-cytokeratin^+^ colon epithelium (Figure 5B). These data validated our proteomic data and supported increased integrin β3 and β-galactosidase expression in 5XFAD mouse colons. As integrin β3 is involved in cell senescence [46], we hypothesized if increased integrin β3 levels were associated with colon senescence in AD. Ki67 contributes to normal cell cycle processes and is a marker of cell cycling but not of resting cells [47,48]. Decreased Ki67 levels and enhanced senescence-related β-galactosidase (SA-β-Gal) activity are widely used to identify senescent cells [47,48,49,50]. In our study, Ki67^+^ cell percentages were significantly decreased in 5XFAD mouse colon crypts (Figure 5B). Moreover, 5XFAD mouse colons showed significantly increased SA-β-gal activities when compared with age-matched controls (Figure 5C). Thus, epithelial cell senescence had occurred in 5XFAD mouse colons. Notably, integrin β3 signals were enriched around Ki67^+^ nuclei, while increased integrin β3 intensity levels corresponded with decreased Ki67 signals in epithelial cells from 5XFAD mouse intestines (Figure 5B).

Both p21- and p16-induced levels robustly reflect cell senescence [19,21]. In our study, p21 mRNA levels were significantly increased in 5XFAD mouse colons when compared with WT controls (Figure 5D), suggesting p21-dependent senescent events. Additionally, cytokines implicated in SASPs, including interleukin-6 (IL-6), tumor necrosis factor-α (TNF-α), C-C Motif Chemokine Ligand 2 (CCL2), plasminogen activator inhibitor-1 (PAI-1), and C-X-C motif chemokine ligand 10 (CXCL10) were significantly induced in 5XFAD mouse colonic tissue when compared with WT controls (Figure 5E). Collectively, strong cell senescence signatures were identified in 5XFAD mouse colons and were associated with increased integrin β3 levels in the epithelium.

### 2.5. Integrin β3 Mediates APP-Induced Senescence in Colon Epithelial Cells

**Figure 6:**
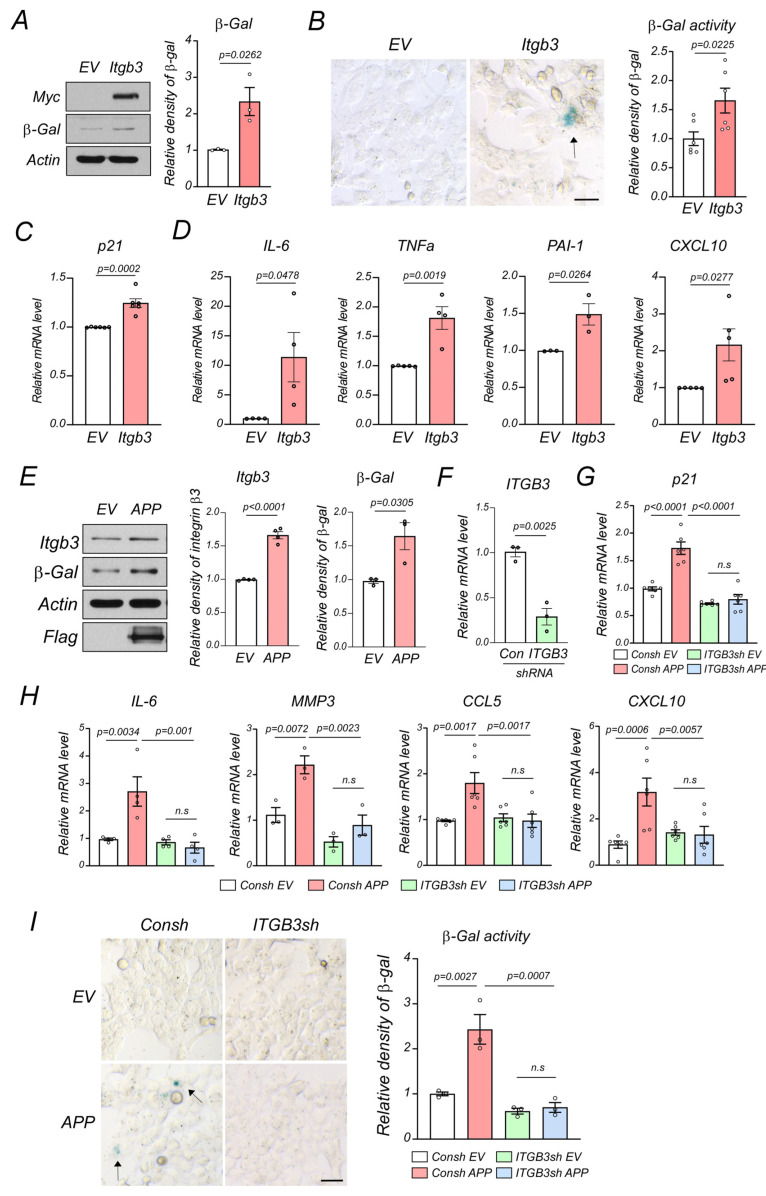
Integrin β3 mediates APP-induced colonic senescence. (**A**) Total protein lysates were harvested from HCT116 cells overexpressing an empty Myc vector (EV) or a Myc-tagged integrin β3 (Itgb3) vector and subjected to western blotting. Representative immunoblots of three independent experiments are shown. Relative β-galactosidase (β-gal) density was quantified. (**B**) Senescence associated β-galactosidase (SA-β-gal) activity was assessed after 48-h overexpression of EV or the Itgb3 vector in HCT116 cells. Representative images of six independent experiments are shown (Scale bar = 25 μm). SA-β-gal activity is indicated by arrow. (**C**) p21- and (**D**)-senescence associated secretory phenotype-related mRNA levels for IL-6, TNFα, PAI-1, and CXCL-10 via a RT-qPCR after the 48-h overexpression of EV or the Itgb3 vector in HCT116 cells. (**E**) Total protein lysates were harvested from HCT116 cells overexpressing the empty flag vector (EV) or a flag-tagged amyloid precursor protein (APP) vector and subjected to western blotting. Representative immunoblots of three independent experiments are shown. (**F**) HCT116 cells stably expressed ITGB3 (short hairpin RNA), shRNA (ITGB3sh), or control shRNA (Consh). ITGB3 mRNA levels were measured via a RT-qPCR to confirm knock-down efficiency. Consh or ITGB3sh HCT116 cells were transfected with the APP vector or EV for 48 h, (**G**) p21- and (H) SASP-related mRNA levels for IL-6, CXCL-10, MMP3 (Matrix Metalloproteinse 3), and CCL5 via a RT-qPCR. (**I**) SA-β-gal activity (indicated by arrow) was assessed in indicated groups (scale bar = 25 μm). Data were expressed as the mean ± standard error of the mean and compared using unpaired Student’s *t*-tests (**A**–**F**) and one-way analysis of variance (**G**–**I**). n.s: no significance.

We next examined if integrin β3 directly induced cell senescence by overexpressing Myc-tagged integrin β3 or Myc-tagged control vectors in HCT116 colonic epithelial cells for 48 h and examined cell senescence marker expression. Integrin β3 overexpression increased β-gal protein levels (Figure 6A), which suggested increased β-gal protein levels in 5XFAD mouse colonic tissue (Figure 3 and Figure 5A) result from elevated integrin β3 levels. Moreover, integrin β3 overexpression enhanced SA-β-gal activity and induced p21- and SASP-associated cytokine expression in colonic epithelial cells (Figure 6B–D). APP is a precursor protein which generates amyloid fragments, is expressed in the colon of human AD patients, and elicits intestinal cell damage [11,33,34,35,36]. In our study, human APP overexpression upregulated integrin β3 and β-galactosidase levels (Figure 6E). This overexpression also induced cell senescence as evidenced by enhanced p21- and SASP-related cytokine expression and SA-β-gal functions (Figure 6F–I). Notably, in APP-overexpressing colonic epithelial cells, integrin β3 knock-down via shRNA significantly reduced SA-β-gal functions, inhibited p21, and suppressed the induction of SASP-related cytokines (Figure 6F–I). Thus, these data suggested that integrin β3 mediated colon senescence in an AD context, and increased integrin β3 levels in 5XFAD mouse colonic tissue, likely accelerated senescence, leading to local inflammation and tissue degeneration.

## 3. Discussion

Gut abnormalities contribute to AD pathogenesis and development; however, detailed mechanisms and cell phenotypes implicated in these abnormalities remain unknown. In our study, for the first time, we identified cell senescence in the gut tissue of transgenic AD mice, concomitant with intestinal inflammation and tissue degeneration. Our unbiased proteomic and biochemical analyses showed that increased integrin β3 levels were implicated in upregulated cell senescence and gut inflammation in AD mouse models. These findings suggest new intestinal inflammation and tissue damage mechanisms in AD.

Persistent and chronic inflammation are prominent traits in AD and are hypothesized to mediate cognitive decline and neurodegeneration [8,17,51]. A recent study reported that chronic mild gut inflammation, induced by low dextran sodium sulfate concentrations, accelerated cognitive dysfunction onset and toxic protein aggregation in a 3xTg AD mouse model [52]. In our study, in 5XFAD and APP KI mouse colons, multiple cytokines and inflammatory cell numbers had age-dependently increased. Our cross-sectional proteomic analysis consistently showed that immune response was the top altered pathway in 5XFAD mice colons with respect to time. Our data suggested that immune cell activation in AD was putatively not limited to brain glial cells but included other tissues, consistent with the concept that inflammatory changes in AD brains are possibly associated with immune alterations in peripheral tissues [53].

There is limited evidence to suggest that the bulk proteome, transcriptome, or metabolome in 5XFAD mice tissue show age-dependent alterations. This could partially explain why less proteins were identified in the colon tissue of 6-month-old mice compared to 3-month-old mice. The goal of conducting proteomics analyses on colon tissues in our study was to identify potential molecular mechanisms that underlie intestinal inflammation and AD-related alterations. These mechanisms likely play a role in the prodromal/early symptomatic stage and across all stages of AD. Thus, our analysis focused only on enriched proteins that consistently changed over time, as depicted in Figure 3. Intriguingly, AD mouse intestines underwent significant cell senescence, accompanied by tissue inflammation. Cell senescence activation-induced SASPs which were manifested by pro-inflammatory cytokine release to neighboring tissue. Chronic SASP-associated inflammation may have then promoted inflammatory environments in the gut, which possibly enhanced gut permeability, immune activation, and gut microbiome composition [54,55]. Additionally, gut microbiota pathogens can upregulate gut and systemic inflammatory reactions, increase intestinal permeability, and contribute to AD-associated neuropathology and cognitive decline [11,17]. Thus, intestinal cell senescence may initialize inflammation and promote the vicious cycle between gut tissue abnormalities and microbiota alterations.

Cell senescence is regulated by intracellular and extracellular signals as well as the extracellular matrix (ECM) [46,56,57]. Integrins are the most common ECM regulators; they are heterodimeric cell surface transmembrane receptors which support signal transduction and cell adhesion [45]. Integrin signaling also regulates many cell functions and is involved in neurological disorders, cancer, and wound healing [58]. Integrin β3 was recently identified as a regulator of p21-dependent cell senescence in human primary fibroblasts [46]. We identified integrin β3 from our proteomics analyses of 5XFAD mice colons and showed its protein levels increased in an age-dependent manner and were correlated with gut inflammation at early AD stages. This observation was supported by integrin β3 overexpression which increased cell senescence markers, whereas integrin β3 knock-down diminished APP-induced cell senescence in colonic epithelial cells. Moreover, p21—but not p16—was significantly induced naturally with enhanced integrin β3 and cell senescence signature levels, consistent with a previous report [46]. In the future, we will determine the impact of gut epithelial integrin β3-mediated cell senescence on in vivo AD-associated pathophysiology in AD animal models.

In summary, by investigating gut tissue integrity and the proteome in AD mice, integrin β3-mediated cell senescence was implicated in gut inflammation and intestinal tissue degeneration in AD models. We provide a new understanding of the molecular resources required for inflammatory responses during AD, and propose integrin β3 as a novel target mediating gut abnormalities in AD.

## 4. Methods and Materials

### 4.1. Reagents and Antibodies

The protein phosphatase inhibitor cocktail (P5726) and protease inhibitor cocktails (8340) were purchased from MilliporeSigma (Berlington, MA, USA). All antibodies are listed here; anti-integrin β3 (Abcam, Cambridge, UK, ab179473), anti-Aβ_1-16_ (6E10) (Biolegend, San Diego, CA, USA) (803015), anti-c-Myc (Santa Cruz Biotechnology, Dallas, TX, USA) (sc-40), anti-β-galactosidase (Proteintech, Rosemont, IL, USA) (15518-1-AP), anti-actin (MilliporeSigma, Burlington, MA, USA) (A1978), anti-pan-cytokeratin (Thermo Fisher Scientific, Waltham, MA, USA, 53-9003-80), anti-Ki67 (Thermo Fisher Scientific, 14-5698-80), anti-Ly6G (Abcam, ab238132), anti-CD3 (Novusbio, Englewood, CO, USA) (NBP2-25186SS), and F4/80 (Proteintech, 28463-1-AP). The senescence β-galactosidase staining kit came from Cell Signaling (Danvers, MA, USA) (9860). Nuclear-fast red aluminum sulfate (0.1%) (1001210500), Alcian blue (1016470500), and hematoxylin and eosin (H&E) stains (HT110216) were supplied by MilliporeSigma.

### 4.2. Cell Culturte

Human colon epithelial cells (HCT116) (American Type Culture Collection, ATCC, Manassas, VA, USA) (CCL-247) were grown in McCoy’s 5A medium (ATCC), 10% fetal bovine serum (Corning), and 100 mg/mL each of streptomycin and penicillin and were cultured in 5% CO_2_ at 37 °C. Cells were used up to passage 14.

### 4.3. AD Animal Model

All animal experiments were conducted in accordance with protocols approved by the Institutional Animal Care and Use Committee of Case Western Reserve University and performed according to the National Institutes of Health Guide for the Care and Use of Laboratory Animals. Sufficient procedures were employed to reduce the pain and discomfort of the mice during the experiments. All mice were maintained under a 12 h/12 h light/dark cycle (light on at 6 AM and off at 6 PM) with ad libitum access to food and water under the ambient temperature at 23 °C and with humidity at 40–60%. The mice were mated, bred, and genotyped in the animal facility of Case Western Reserve University. All mice used in this study were maintained on a C57BL/6J (Strain #000664, The Jackson Laboratory, Bar Harbor, ME, USA) background. 5XFAD transgenic mice (Tg (APPSwFlLon, PSEN1*M146L*L286V) 6799Vas, strain #034840-JAX) breeders were purchased from Jackson Laboratory. APP^NL-G-F^ knock-in breeder mice (stock # RBRC06344) were obtained from Japan Riken BioResource Research Center. All mice are genotyped before experiments based on the genotyping protocol the vendors provided.

### 4.4. Label-Free Proteomics

Each frozen mouse colon sample (*n* = 3 mice per group/time point) was collected in a 1.5mL tube containing 300 µL of 2% SDS and protease inhibitor cocktail (Sigma, St. Louis, MO, USA). The samples were incubated on ice for 30 min and then sonicated with a probe sonicator at 50% amplitude followed by vortexing; this cycle was repeated 4 times with samples sitting on ice between each round. Following lysis, the samples were processed using a filter-aided sample preparation cleanup protocol with Amicon Ultra MWCO 3K filters (Millipore, Billerica, MA, USA). Samples were reduced and alkylated on the filters with 10 mM dithiothreitol (Acros, Fair Lawn, NJ, USA) and 25 mM iodoacetamide (Acros, Fair Lawn, NJ, USA), respectively, and then concentrated to a final volume of 40 µL in 8 M Urea. Protein concentration was measured using the Bradford method according to the manufacturer’s instructions (Bio-Rad, Hercules, CA, USA).

Following reduction and alkylation, the total protein (10 mg) was subjected to enzymatic digestion. The urea concentration was adjusted to 4 M using 50 mM Tris pH 8, and then proteins were digested with mass spectrometry-grade lysyl endopeptidase (Wako Chemicals, Richmond, VA, USA) in an enzyme/substrate ratio of 1:40 for 2 h at 37 °C. The urea concentration was further adjusted to 2M using 50mM Tris pH 8, and the lysyl peptides were then digested with sequencing-grade trypsin (Promega, Madison, WI, USA) in an enzyme/substrate ratio of 1:40 at 37 °C overnight. Finally, the samples were diluted in 0.1% formic acid (Thermo Scientific, Rockford, IL, USA) before LC-MS/MS analysis.

The peptide digests (320 µg) were loaded onto a column in an 8 µL injection volume with blanks in between for a total of four LC/MS/MS runs. The resulting data were acquired with an Orbitrap Velos Elite mass spectrometer (Thermo Electron, San Jose, CA, USA) equipped with a Waters nanoACQUITY LC system (Waters, Taunton, MA, USA). The peptides were desalted on a trap column (180 μm × 20 mm, packed with C18 Symmetry, 5 μm, 100Å, Waters, Taunton, MA, USA) and subsequently resolved on a reversed-phase column (75 μm × 250 mm nano column, packed with C18 BEH130, 1.7 μm, 130 Å (Waters, Taunton, MA, USA). Liquid chromatography was carried out at an ambient temperature at a flow rate of 300 nL/min using a gradient mixture of 0.1% formic acid in water (solvent A) and 0.1% formic acid in acetonitrile (solvent B). The gradient ranged from 4 to 44% solvent B over 210 min. The peptides eluting from the capillary tip were introduced into the nanospray mode with a capillary voltage of 2.4 kV. A full scan was obtained for the eluted peptides in the range of 380–1800 atomic mass units followed by twenty-five data dependent MS/MS scans. The MS/MS spectra were generated by collision-induced dissociation of the peptide ions at a normalized collision energy of 35% to generate a series of b- and y-ions as major fragments. In addition, a one-hour wash was included between each sample. The proteins were identified and quantified using PEAKS 8.5 (Bioinformatics Solutions Inc., Waterloo, ON, CA, USA). The proteomic datasets were submitted to figureshare (https://figshare.com/) with https://figshare.com/articles/dataset/Mass_spec_proteins_original_file_xls/22203199 (accessed on 2 March 2023).

### 4.5. Bioinformatics

DisGeNET: Gene-disease associated data with AD (CUI: C0002395) were retrieved from DisGeNET v6.0 (http://www.disgenet.org/) (accessed on 2 March 2023), Integrative Biomedical Informatics Group GRIB/IMIM/UPF(April, 2022) [59].

Enrichment pathway analysis: PANTHER overrepresentation tests with Reactome pathway annotations (Fischer’s exact tests and false discovery rate (FDR) correction FDR *p* < 0.05) were conducted on upregulated/downregulated proteins (5XFAD/WT: 0.5 ≥ fold change (FC) ≥ 2) [60,61]. Database effect sizes were analyzed in VolcaNoseR (https://huygens.science.uva.nl/VolcaNoseR/) (accessed on 2 March 2023) [62].

Functional enrichment analyses: We used Database for Annotation, Visualization and Integrated Discovery (https://david.ncifcrf.gov/) (accessed on 2 March 2023) [63,64] to visualize functionally grouped networks. Altered proteins (5XFAD/WT, 2-fold upregulated or downregulated) against gene ontology (GO) terms and Reactome reference sets were tested to generate enriched terms using two-sided hypergeometric tests using Bonferroni step-down corrections. Enriched terms with *p* ≤ 0.01 values are provided.

### 4.6. Knowledge Graph (KG) Predictions of AD-Associated Genes

KG construction: To construct the KG graph, we integrated multiple entities and relationships from different phenotypic and genotypic databases, including Gene Set Enrichment Analysis (GSEA), Mouse Genome Informatics (MGI), Gene Ontology Annotation (GOA), Genotype-Tissue Expression (GTEx), Phenomebrowser databases, Human Phenotype Ontology (HPO) database, DrugBank, and TreatKB. The KG was composed of 1,395,766 interactions between 65,298 entities (Figure from Section 2.2).

GSEA database of gene-phenotype interactions: This database comprised 16,325 biomedical entities and 149,040 relationships from the GSEA database. This comprehensive computational platform provides significant and concordant interactions between two biological states, e.g., genes–phenotypes [65,66].

MGI database for gene-related mammalian phenotype (MP) interactions: This database comprised 26,917 biomedical entities and 194,686 relationships. Data from the MGI [67] provided information on laboratory mouse genetics, genomics, and biology to promote human health and disease studies.

GOA database of gene-related GOA interactions: Comprising 32,207 biomedical entities and 204,862 relationships, GOA provides annotations for the UniProt Knowledgebase using standardized GO vocabulary [68].

GTEx database for gene-related UT interactions: Comprising 16,630 biomedical entities and 539,845 relationships, GTEx data resources and the tissue bank provide information on genetic variants and gene expression in multiple human tissue samples in different individuals [69].

Phenomebrowser database for drug-related HP and MP interactions: Composed of 5795 biomedical entities and 212,135 relationships, Phenomebrowser combines phenotypic connections with biomedical concepts [70] and provides drug–phenotype datasets, including MP annotations [71] and human phenotype (HP) ontology-associated drugs [72].

HPO database for disease-related HP interactions: With 13,956 biomedical entities and 87,154 relationships, HPO provides a standardized vocabulary of phenotypic abnormalities in human disease [72].

DrugBank of drug–drug target interactions: Comprising 2350 biomedical entities and 5280 relationships, DrugBank provides comprehensive drug and drug target information [73].

TreatKB of drug–disease interactions: We previously developed [74,75] natural language processing techniques to extract drug–treatment–disease interactions from patients’ records from the Food and Drug Administration (FDA) Adverse Event Reporting System, FDA drug labels, MEDLINE abstracts, and clinical trials. We extracted 2764 drug-disease pairs from TreatKB.

Prioritization algorithm: We applied processed KGs to KG-predict what we recently developed [42,43]. Our KG predictions included embedding and predicting modules. The embedding module used the KG as an input and learned the low-dimensional embedding of entities and relations. For a disease node, the embedding module aggregated information from the disease identifier, topological structures of disease neighborhoods (e.g., genes, drugs, and phenotype annotations), and semantic relationships between the disease and its neighbors to learn disease embedding. Once learned, the predicting module concatenated entity and relationship embeddings and used three operations to extract topological and semantic information to generate linked predictions. When inputting a disease entity (e.g., AD) and “Disease-Associate-Gene”, predicting modules generated rich heterogeneous interactions between the entity and relation embedding to determine novel disease–gene interactions.

### 4.7. Quantitative Real Time PCR (RT-qPCR)

Using TRIzol (Invitrogen, Waltham, MA, USA) (15596-026) and RNeasy Mini Kits (QIAGEN, Germantown, MD, USA) (74104), we isolated total RNA from mouse colon tissue and cells, respectively, and 1 µg was used for cDNA synthesis using QuantiTect Reverse Transcription kits (QIAGEN, 205311). Next, we conducted a qRT-PCR using SYBR Green Master Mix (Thermo Fisher Scientific, A25743) and reactions were processed using QuantStudio 3 Real-Time PCR Instrumentation (Thermo Fisher Scientific). For samples, three replicates were performed, and values from the replicates were normalized against glyceraldehyde 3-phosphate dehydrogenase (GAPDH) cDNA using the 2^−ΔΔCT^ approach. The following primers were used (Table 1).

### 4.8. Western Blotting

Using Bradford assays (Bio-Rad Laboratories, Hercules, CA, USA), protein concentrations were determined, after which 15–25 µg was added to a 5× Laemmli buffer, samples were boiled for 5 min, and proteins underwent SDS–polyacrylamide gel electrophoresis. Separated proteins were electrophoretically transferred to nitrocellulose membranes (Bio-Rad Laboratories), blocked in 5% non-fat milk in Tris-buffered saline plus 0.1% Tween 20 (TBST) for 1 h, and probed overnight with the aforementioned primary antibodies. After washing 3× in TBST, secondary anti-rabbit or anti-mouse IgG (Catalog numbers: 31430/31460, Thermo Fisher Scientific) antibodies were added, and membranes were incubated at room temperature (RT) for 1 h, washed, and visualized using enhanced chemiluminescence. Representative blots were cropped for figures.

### 4.9. Constructs and Transfections

pCAX FLAG APP (Catalog number: 30154) and pcDNA3.1-beta-3 (Catalog number: 27289) vectors were supplied by Addgene (Watertown, MA, USA). Using TransIT-2020 transfection reagent (Mirus Bio, LLC, Madison, WI, USA), cells were transfected according to kit protocols.

### 4.10. H&E Staining

At RT, frozen sections were left for 1 h to thaw and dry out and were then fixed in acetone for 15 min. Next, sections were serially immersed in 99%, 80%, and 70% ethanol (all for 3 min) and washed in distilled deionized water (3 min). Sections were then stained in 10% hematoxylin for 5 min, washed for 10 min in running water, stained in 1% eosin for 5 min, and rewashed for 3 min in distilled deionized water. Sections were then serially dehydrated in 70%, 80%, 99% ethanol, and xylene (3 × 3 min for each) before mounting. A Keyence all-in-one microscope BZX710 (KEYENCE, Osaka, Japan) was used to capture H&E images and for imaging in several assays below.

### 4.11. Alcian Blue Staining

To thaw and dry out, frozen sections were left for 1 h at RT and rinsed in water for 10 min. Using 1% Alcian blue in 3% acetic acid, sections were stained for 30 min, rinsed in running water for 10 min, stained in 0.1% nuclear fast red aluminum sulfate solution for 60 s, and rinsed in running water for 10 min. Sections were serially dehydrated in 50%, 70%, 95%, 100% ethanol (1 min each time), and xylene (3 × 5 min for each) before mounting. Images were captured as described.

### 4.12. Immunohistochemistry (IHC)

Deeply anesthetized mice were transcardially perfused using 4% paraformaldehyde in phosphate-buffered saline (PBS). Gut tissue was removed and post-fixed overnight in 4% paraformaldehyde at 4 ℃ and equilibrated in 30% sucrose. Frozen 20 μm coronal colon sections were hydrated, immersed in 3% hydrogen peroxide in methanol, and incubated for 1 h with 5% normal goat serum at RT. Next, in a humidified chamber, mouse anti-Ly6G (1:200, Abcam), anti-CD3 (1:200, Novusbio), and anti-F4/80 (1:200, Proteintech) antibodies were incubated with sections overnight at 4 ℃. After washing in PBS, Ly6G, CD3, and F4/80IHC localization were determined using a HRP/DAB kit (Catalog number: 20774 and 20775, MilliporeSigma). Images were captured as described. We quantified immunostaining using ImageJ (NIH). For all sections, the same threshold settings and image exposure times were used.

### 4.13. Immunofluorescence

Frozen 20 μm mouse colon sections were stained with anti-integrin β3 (1:200, Abcam), anti-pan-cytokeratin (1:200, Thermo Fisher Scientific), ani-Ki67 (1:200, Thermo Fisher Scientific) antibodies and incubated in a humidified chamber overnight at 4 °C. Then, after washing in PBS, sections were incubated with secondary antibodies conjugated to goat anti-mouse/rabbit/rat Alexa Fluor 488/568/647 (1:500; Thermo Fisher Scientific) for 2 h at RT.

After counterstaining with Hoescht33342 (1:10,000; Thermo Fisher Scientific, catalog number: 62249), sections were mounted in mounting medium (Dako, Santa Clara, CA, USA). A FV3000 confocal microscope (Olympus, Tokyo, Japan) was used to capture images and immunostaining was quantified using ImageJ. For all sections, the same threshold settings and image exposure times were used.

### 4.14. Total Lysate Preparations

Cells washed in cold PBS (pH 7.4) were cooled in ice for 30 min in a total lysis buffer (50 mM Tris-HCl (pH 7.5), 150 mM NaCl, and 1% Triton X-100, plus protease and phosphatase inhibitor cocktails). Mouse colon tissues homogenized in a RIPA buffer (10 mM Tris-HCl (pH 7.5), 150 mM NaCl, 1 mM EDTA, 1% Triton X-100, 0.1% SDS, and 0.1% sodium deoxycholate) were cooled in ice for 30 min. To generate total lysate supernatants, samples were centrifuged at 12,000× *g* at 4 °C for 10 min.

### 4.15. β-Galactosidase Activity Assays

Using kit protocols (Cell Signaling, 9860S), β-galactosidase activity was measured. Briefly, cells grown in a 12-well plate were washed in PBS and immersed in 1× fixing solution (provided in kit) at RT for 15 min. After washing twice in PBS, a β-galactosidase stain was added to cells and samples incubated overnight at 37 °C. Cells were imaged as described.

### 4.16. RNA Interference

Control short hairpin RNA (shRNA) and ITGB3 shRNA (TRCN0000003237) molecules were supplied by MilliporeSigma. Cells infected with lentiviral particles expressing shRNAs for 48 h were selected using 2 μg/mL puromycin (Corning Inc., Corning, NY, USA) to select stable ITGB3 knock-down cell lines.

### 4.17. Statistical Analyses

Using published or our pilot data, sample sizes were determined using power analyses. Cell culture investigations had at least three independent replications; we ensured randomization and blinding in animal studies, and for imaging analyses, observers blinded to study groups performed quantitation analyses.

GraphPad Prism 9.0 was used to process data and we used Student’s *t*-tests to compare data between two groups. For three or more independent group comparisons, one-way analysis of variance, followed by Tukey’s post hoc tests were used. Data were represented as the mean ± standard error of the mean, statistical information is shown in figure legends, and *p* < 0.05 was statistically significant.

## Figures and Tables

**Table 1:** Primers used in quantitative real time PCR.

Mouse		Human	
GAPDH	s-GACTTCAACAGCAACTCCCAC	GAPDH	s-GCGAGATCCCTCCAAAATCAA
	as-TCCACCACCCTGTTGCTGTA		as-GTTCACACCCATGACGAACAT
P21	s-CGAGAACGGTGGAACTTTGAC	P21	s-TCACTGTCTTGTACCCTTGTGC
	as-CAGGGCTCAGGTAGACCTTG		as-GGCGTTTGGAGTGGTAGAAA
IL-6	s-GCTACCAAACTGGATATAATCAGGA	IL-6	s-CAGGAGCCCAGCTATGAACT
	as-CCAGGTAGCTATGGTACTCCAGAA		as-GAAGGCAGCAGGCAACAC
TNFα	s-GCCCAGGCAGTCAGATCATCT	TNFα	s-GCCCAGGCAGTCAGATCATCT
	as-TTGAGGGTTTGCTACAACATGG		as-TTGAGGGTTTGCTACAACATGG
CCL2	s-CATCCACGTGTTGGCTCA		Not tested
	as-GATCATCTTGCTGGTGAATGAGT		
PAI-1	s-GACACCCTCAGCATGTTCATC	PAI-1	s-AGCTCCTTGTACAGATGCCG
	as-AGGGTTGCACTAAACATGTCAG		as-ACAACAGGAGGAGAAACCCA
CXCL10	s-GCTGCCGTCATTTTCTGC	CXCL10	s-GAAAGCAGTTAGCAAGGAAAGGT
	as-TCTCACTGGCCCGTCATC		as-GACATATACTCCATGTAGGGAAGTGA

## Data Availability

The datasets generated and/or analyzed during the current study are available from the corresponding author on reasonable request.
